# The value of intellectual structural imbalance in the differentiation of autism spectrum disorder and attention deficit hyperactivity disorder

**DOI:** 10.3389/fpsyt.2025.1610278

**Published:** 2025-08-14

**Authors:** Dan-Yang Zhang, Qiu-Hong Wei, Bin-Yue Hu, Dan Ai, Yu Zhang, Yuan Ding, Ting Yang, Jie Chen, Li Chen, Yuan Wu, Hong-Yu Chen, Xue-Li Xiang, Qiu-Hong Mou, Ting-Yu Li

**Affiliations:** ^1^ Children Nutrition Research Center, Children’s Hospital of Chongqing Medical University, Chongqing Key Laboratory of Child Neurodevelopment and Cognitive Disorders, Ministry of Education Key Laboratory of Child Development and Disorders, National Clinical Research Center for Child Health and Disorders, Chongqing, China; ^2^ Department of Primary Child Health Care, Children’s Hospital of Chongqing Medical University, Chongqing, China

**Keywords:** autism spectrum disorder, attention deficit hyperactivity disorder, Wechsler intelligence scale of children (C-WISC), intellectual structures, comorbidity

## Abstract

**Background:**

The study delves into the intricate task of differentiating intellectual structures among children diagnosed with the high-functioning Autism Spectrum Disorder (HF-ASD), Attention Deficit Hyperactivity Disorder (ADHD), or comorbidity (ASD+ADHD), aiming to assist in their clinical differentiation, with the goal of refining clinical diagnoses and developing targeted therapeutic interventions.

**Methods:**

The study included 200 outpatients aged 6.5–13.0 years (total Intelligence Quotient (IQ) 70–130) at the Children’s Hospital of Chongqing Medical University, and categorized into HF-ASD (n=91), ADHD (n=47), and comorbidity ASD+ADHD (n=62) groups. We utilized the Chinese Wechsler Intelligence Scale for Children (C-WISC) as the primary assessment tool, supplemented by additional diagnostic measures. Besides, we used SPSS 25.0 to assess the subtest scores and differences.

**Results:**

The comorbidity group had lower total IQ than the other two groups (*p<0.001*). The verbal IQ(VIQ) were lower than the performance IQ(PIQ) in HF-ASD (*p=0.017*) and comorbidity (*p=0.007*) groups. They also scored higher on perceptual organization subtests particularly in Block Design and Object Assembly than the ADHD group. The ADHD group showed higher VIQ than PIQ (*p=0.020*). The ADHD group’s scores for working memory subtests were lower than in the HF-ASD group. The respective peak scores for the HF-ASD and comorbidity groups were in Block Design (45%,43%) and Object Assembly (30%,37%) and valleys in Picture Completion (52%,24%), Information (HF-ASD 24%), and Arithmetic (comorbidity 42%).

**Conclusion:**

The peak-valley difference in the ADHD group (~2 standard deviations) was smaller than in the HF-ASD and comorbidity groups (~3 standard deviations), and this characteristic could help differentiate between HF-ASD, ADHD, and both together.

## 1 Introduction

Autism Spectrum Disorders (ASD) is a complex neurodevelopmental condition marked by two primary symptoms: deficient social communication skills and limited interests accompanied by repetitive, stereotyped behaviors. The prevalence of the disease globally is approximately 1% (1) and it has been increasing in recent years (2). Because ASD is clinically heterogeneous and can be divided into High-Functioning ASD (HF-ASD) (IQ≥70) and Low-Functioning ASD(LF-ASD) (IQ<70) based on intelligence quotient (IQ) (3, 4). Among individuals with HF-ASD exhibit normal intelligence and milder social challenges, often displaying highly selective focus (5).Previous studies have shown that they often show symptoms of attention deficit and hyperactivity disorder (ADHD) (6). ADHD, one of the most prevalent neurodevelopmental disorder, is characterized by inattention, hyperactivity, and impulsivity, affecting approximately 7% of children globally (7, 8). ADHD is a common comorbidity of ASD, with coexistence rates ranging from 40% to 70% (9–11). Both disorders often present with inattention as the primary concern in clinical settings, and their overlapping symptoms can easily lead to confusion, complicating their distinction (12, 13). A survey of delayed ASD diagnosis among children and adolescents found that a significant proportion of ASD cases were misdiagnosed or underdiagnosed, with ADHD emerging as the most common misdiagnosis (14, 15). Therefore, failing to accurately differentiate between the two disorders may result in inappropriate treatment, delayed intervention, and worsened social functioning impairment (16, 17).

At present, the Wechsler Intelligence Scale (WISC) have emerged as critical tools for distinguishing these disorders (18, 19). Previous studies have shown that the difference between verbal Intelligence Quotient (VIQ) and Performance Intelligence Quotient (PIQ) helps distinguish ASD from ADHD individuals. Among them, the VIQ of children with ASD was significantly lower than that of PIQ (20, 21) Conversely, some studies of intellectual ability reported no VIQ and PIQ differences in ASD (22). Besides, A comparison study between children with ASD and ADHD found that those with HF-ASD scored significantly lower in verbal comprehension, vocabulary, and understanding, but higher in block design compared to children with ADHD (23). This unbalanced pattern of intelligence is attributed to the neurodiversity of its development. As outlined in the *British Medical Bulletin*, the psychological definition of neurodiversity reflects the diversity of individual cognitive abilities, in which there are large, significant differences between the peaks and valleys of the profile. Individuals with neurodiversity exhibit cognitive profiles where abilities span ≥2 standard deviations(SD) within a normal distribution (24). The scores of WISC provided clear guidance on the level of difference between strengths and weaknesses that is typical or of clinical significance, and were used to support the diagnosis of neurodevelopmental disorders (25). Emerging evidence suggests such cognitive development patterns may better differentiate neurodevelopmental profiles than conventional metrics (21, 26). This was confirmed by the study of Koyama et al. (23).

Previous studies have primarily focused on the analysis of intellectual characteristics in children diagnosed solely with HF-ASD and ADHD, while research on children with both ASD and ADHD is relatively scarce. Additionally, it has been noted that there are differences in the WISC scale scores between the two conditions. However, few studies have conducted in-depth analyses of the intellectual patterns combining these three disorders. This methodological gap impedes the identification of these disorder cognitive traits, which are essential for refining diagnostic precision and developing targeted interventions. Therefore, resolving this diagnostic ambiguity holds urgent clinical significance. The present study seeks to investigate the intellectual structure imbalance in children with ASD and ADHD, as measured by the Wechsler Intelligence Scale. By analyzing cognitive paradigms linked to neurodiversity, this research aims to establish a foundation for clinical differential diagnosis and targeted interventions.

## 2 Methods

### 2.1 Participants and procedure

The study is a single-center case-control analysis conducted at the Children’s Hospital Affiliated with Chongqing Medical University from 2023 to 2024.This cross-sectional study recruited 421 children and adolescents aged 6.5–13.0 years, with primary clinical presentations of attentional deficits. Participants underwent a multi-phase diagnostic evaluation to stratify neurodevelopmental profiles. Inclusion criteria mandated comprehensive medical documentation and baseline cognitive assessment using the Chinese Wechsler Intelligence Scale for Children (C-WISC)(total IQ>70) (27, 28), Social Responsiveness Scale (SRS),Autism Diagnostic Observation Schedule (ADOS)(excluding the ADHD group), Infant-Junior High School Student Social Life Ability Scale (S-M Scale), and Vanderbilt ADHD Rating scales. The final analytical cohort comprised 200 participants after exclusions for missing diagnostic information (22%, n=49), incomplete diagnostic scale(21%, n=47), uncertain diagnosis (5%, n=10),diagnosed with other diseases(10%,n=22), IQ < 70 (17%, n=38), age < 6 years (10%, n=22), and coexisting other neurodevelopmental disorders (15%, n=33). Diagnosed by two developmental-behavioral pediatric experts following the Diagnostic and Statistical Manual of Mental Disorders, Fifth Edition (DSM-5) criteria for ASD, ADHD, and ASD + ADHD (7, 29, 30). Inter-rater reliability was evaluated using Bland-Altman analysis to quantify agreement between ADOS and SRS scores, the scatters are basically within the 95% agreement interval (within 1.96 SDs) (31), indicating that the consistency was considered acceptable. Discrepancies were resolved through panel review. Strict data integrity checks ensured exclusion of ambiguous or incomplete cases, enhancing diagnostic homogeneity. In addition, the inclusion method of this study is included retrospectively. And the detailed schematic diagram for specific clinical diagnosis groupings is provided in **Appendix Table 1**. This study has been approved by the Ethics Committee of the Children’s Hospital Affiliated with Chongqing Medical University (2024305).

### 2.2 Assessment tools

Standardized assessment procedures, including the same set of evaluations, were followed for all participants.

#### 2.2.1 Chinese Wechsler intelligence scale for children

Devised by Hunan Medical University, the C-WISC is suitable for children aged 6 to 16 years. This scale has undergone rigorous cultural adaptation including linguistic modifications to reflect Mandarin semantic structures and norming processes specific to urban Chinese populations. Its norming procedure includes stratified sampling based on age, gender, urban-rural distribution, and parents’ educational level, strictly following the WISC-R’s operational and scoring standards, with IQ scores based on a mean of 100 and a standard deviation of 15. Its reliability and validity are close to those of the original version (WISC-R) and meet statistical requirements (28). It consists of a Verbal Scale (Vs) comprising Information, Similarities, Arithmetic, Vocabulary, and Digit Span subtests, and a Performance Scale (Ps) comprising Picture Completion, Picture Arrangement, Block Design, Object Assembly, and Coding subtests. Verbal IQ (VIQ) is derived from the Vs scores, and performance IQ (PIQ) is derived from the Ps scores. The full-scale IQ is based on the combined Vs and Ps. Subtests were grouped into Verbal Comprehension (Information, Similarities, Vocabulary), Perceptual Organization (Picture Completion, Picture Arrangement, Block Design, Object Assembly), and Working Memory (Arithmetic, Digit Span, Coding) factors. The IQ range for normal children is 70-130. An IQ below 70 is considered intellectual disability. If a child has both an IQ below 70 and ASD, they are diagnosed with low-functioning ASD. In addition to that, a VIQ-PIQ difference > 15 indicated clinically significant intellectual imbalance. Internally, this refers to the difference between high and low scores within operational or linguistic subdomains. Overalls, it encompasses the variation between the highest and lowest scores across the entire battery of tests.

#### 2.2.2 Autism diagnostic observation schedule

The Chinese version of ADOS was used. This version has been validated as having good reliability and practical validity (32).It is considered the gold standard for assessing ASD symptoms in one-year-old individuals and older (33–35). It evaluates social interaction, communication, stereotypic behaviors, interests, and imagination. Scores indicate the severity of the autistic symptoms. The scale consists of different modules and differs in terms of diagnostic threshold scores for autism and autism spectrum disorders (ASD).

#### 2.2.3 Vanderbilt ADHD rating scale

The scale includes parent rating scales, teacher rating scales and Rating scale-IV(RS-IV). It is used to assess ADHD symptoms, functional impairment and treatment response in children and adolescents (36, 37). It also aids in differentiating ASD + ADHD from other neurodevelopmental disorders. A positive diagnosis criterion may encompass the following medical and professional aspects: a. Number of Symptoms: A minimum of six symptoms must meet the positive criteria within either the inattentive or hyperactive-impulsive domains. b. Duration of Symptoms: These symptoms must persist for at least six months and be more pronounced than what is typically expected for the child’s developmental level. c. Occurrence of Symptoms: The symptoms must manifest in at least two settings (e.g., home and school). d. Functional Impairment (functional impairment scales): These symptoms must interfere with the child’s normal functioning in family, school, or work environments. Although different studies have reported varying results regarding the validity and reliability analysis, they generally indicate that the scale has high consistency and practicality for the diagnosis of ADHD (38).

#### 2.2.4 Social responsiveness scale

This scale assesses ASD features across age groups, quantifying social awareness, cognition, communication, motivation, and restricted interests/repetitive behaviors (39, 40). Scores ≥65 indicate positive ASD features, with higher scores reflecting greater social impairment (41). The reliability and validity of this scale in the ASD population aged >4 years are 0.946 and 0.958, respectively, demonstrating good applicability (42).

#### 2.2.5 Infant-junior high school student social life ability scale

This scale evaluates self-reliance skills, motor skills, academic performance, interpersonal skills, collaborative tasks, and self-management, aiding in identifying social interaction impairments in children with ASD. In our country, the reliability of this scale is 0.98, and its validity is over 95% (43). Scores ≤ 9 (standard score) indicate that the adaptive ability may be suspiciously abnormal or deficient (44).

### 2.3 Statistical analysis

Data were analyzed using SPSS Statistics for Macintosh, Version 25.0 (SPSS Inc., Chicago, IL, USA). In our study, normally distributed continuous variables are reported as the mean ± standard deviation (`x± s). To compare multiple groups of these variables, we conducted Repeated-measures ANOVA, with *post-hoc* Least Significant Difference (LSD) tests multiple comparisons, which were employed to evaluate cognitive strengths and weaknesses within each diagnostic group. For non-normally distributed continuous variables, data are presented as the median and range, and group comparisons were carried out using the Kruskal-Wallis H test, with Dunn’s test utilized for pairwise analyses, and applied Bonferroni correction for multiple comparisons to minimize the risk of Type I errors (false positives). Furthermore, we conducted paired t-tests to compare Verbal Intelligence Quotient (VIQ) and Performance Intelligence Quotient (PIQ) scores between children diagnosed with different conditions. Categorical variables are expressed in terms of the number of cases (N) and percentages (%), and within-group comparisons of these data were analyzed using the chi-square test. Across all analyses, a *p*-value of less than 0.05 (*p*< 0.05) was deemed statistically significant.

## 3 Results

### 3.1 Demographics

The study included 200 children aged 6.5–13.0 (mean: 7.499 ± 1.064) years treated at the specialist outpatient department of the Children’s Hospital of Chongqing Medical University between 2023 and 2024. These included the HF-ASD (*n* = 91), ADHD (*n* = 47), and ASD + ADHD (*n* = 62) groups (**Table 1**).Additionally, We also analyzed the results of relevant scales used for auxiliary diagnosis and observed significant differences among children with different diagnoses in terms of Infant-Junior High School Student Social Life Ability Scale (S-M Scale)(*p<0.001*), Social Responsiveness Scale (SRS)(*p<0.001*), Vanderbilt ADHD Diagnostic Rating Scale(*p<0.001*). Furthermore, it is evident that children with ASD + ADHD scored lower on the S-M scale, while their scores on the SRS were higher than in the HF-ASD group.

**Table 1:** Neuropsychological characteristics of the participants.

Item	Group			χ2(F)	*p*
	HF-ASD	ASD+ADHD	ADHD		
Gender (n, %)					
Female	19(21.111)	6(9.524)	11(23.404)	4.646	p=0.200*
Male	71(78.889)	57(90.476)	36(76.596)		
FSIQ	98.370 ± 14.200	84.080 ± 17.480	86.700 ± 11.260	20.054	p<0.001**
S-M Scale	9.760 ± 0.480	9.100 ± 1.100	9.470 ± 0.720	11.089	p<0.001**
SRS Scale	67.100 ± 17.420	79.910 ± 22.600	47.670 ± 6.550	25.849	p<0.001**
ADOS Scale (refer to Appendix Table 2)					
ADHD rating scale (n, %)					
RS-IV(Rating scale-IV)	Positive 12(13.333)	56(88.889)	40(85.106)	123.673	p<0.001**
	Negative78(86.667)	7(11.111)	7(14.894)		
Vanderbilt Parent Rating Scale	Positive 9(10.000)	58(92.063)	40(85.106)	139.358	p<0.001**
	Negative81(90.000)	5(7.937)	7(14.894)		
Vanderbilt Teacher Rating Scale	Positive 11(12.222)	55(87.302)	43(91.489)	134.765	p<0.001**
	Negative79(87.778)	8(12.698)	4(8.511)		

Data are presented as mean ± standard deviation (SD) or N (%). *HF-ASD* High-Functioning Autism Spectrum Disorder, *ADHD* Attention Deficit Hyperactivity Disorder, *ASD+ADHD* comorbidity, *FSIQ* Full Scale Intellectual Quotient, *S-M Scale* Infant-Junior High School Student Social Life Ability Scale, *SRS* Social Responsiveness Scale, *ADOS* Autism Diagnostic Observation Schedule. * p<0.05, **p<0.01.

### 3.2 Comparison of full-scale intellectual quotient

Significant differences were observed between groups (*p<0.001*), with the ASD + ADHD group having the lowest total IQ and the HF-ASD group the highest (**Table 1**).

### 3.3 Comparison of VIQ and PIQ

Paired samples *t*-tests showed that the ADHD group had significantly higher Verbal Intelligence Quotient (VIQ) than Performance Intelligence Quotient (PIQ) (*t* = 2.413, *p= 0.020*), differing by about 5 points (0.3 SDs). In contrast, the HF-ASD and ASD + ADHD groups had significantly lower VIQ than PIQ, differing by about 4 points (**Appendix Figure 1**).

### 3.4 Subtest scores and peak-valley differences

**Figure 1:**
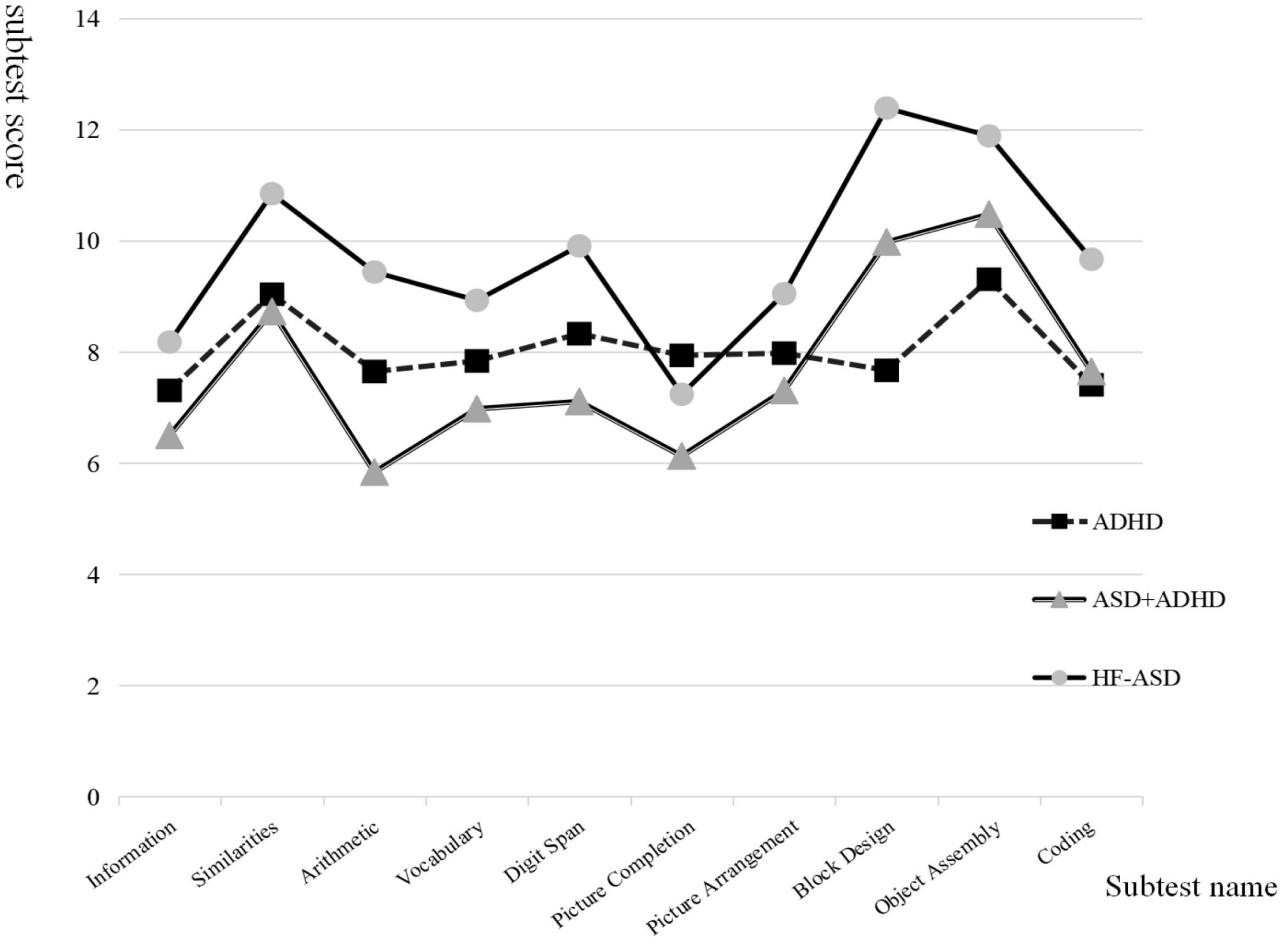
Comparison of score between Children with HF-ASD, ADHD, ASD+ADHD. *HF-ASD* High-Functioning Autism Spectrum Disorder, *ADHD* Attention Deficit Hyperactivity Disorder, *ASD+ADHD* comorbidity.

Kruskal-Wallis tests revealed significant differences in all subtests across groups (*p < 0.001*, SD=3). *Post hoc* comparisons showed that the HF-ASD (12.39 and 9.98 points) and ASD + ADHD (11.89 and 10.48 points) groups scored significantly higher on Block Design and Object Assembly than the ADHD group (7.67 and 9.31points). The ADHD group(7.41 points) scored significantly lower on Coding than the HF-ASD group and ASD + ADHD group(9.44 and 7.65 points)(**Figure 1**). The HF-ASD and ASD + ADHD groups showed greater score variability, with peaks in Block Design and Object Assembly and valleys in Picture Completion and Information. In contrast, the ADHD group showed more stable scores, with peaks in Object Assembly and Classification and a valley in Coding. Then, an analysis of the top three highest and lowest proportions of subtest scores for children with different diagnoses revealed that the highest scores (peaks) of 45% of children with HF-ASD and 43% of those with ASD + ADHD were in Block Design. The respective peak rates in Object Assembly were 30 and 37%. Conversely, their respective lowest score (valleys) rates were >50% and 24% in Picture Completion. Furthermore, 42% of the ASD +ADHD group scored lowest on Arithmetic. In contrast, among children with ADHD, only 6% had their peaks in Block Design, while the peaks of 45% were in Object Assembly and 37% in Similarities. For valleys, 33% of children with ADHD scored lowest on Coding (**Table 2**). Finally, we used the subtest scores of the C-WISC as the x-axis and the SRS scores as the y-axis, we analyzed their correlation. The results revealed a negative correlation between SRS scores and most C-WISC subtests, indicating that higher subtest scores were associated with lower SRS scores (i.e., reduced social impairment). In contrast, Block Design and Object Assembly subtests demonstrated a positive correlation with SRS scores, meaning higher scores on these subtests corresponded to higher SRS scores (i.e., greater social deficits).Subsequently, we used partial correlation analysis to analyze the correlation between SRS scores and block design, with total IQ, verbal IQ, and age as control variables. The results showed that after excluding confounding factors, there was still a correlation between block design and SRS scores(*p=0.237*). (**Appendix Figure 3**).

**Table 2:** Comparison of score between Children with HF-ASD, ADHD, ASD+ADHD.

Characteristics	The top three categories in terms of the proportion of HF-ASD	HF-ASD N (%)	ASD+ADHD N (%)	ADHD N (%)
Peak	Block Design	40 (45%)	27 (43%)	3 (6%)
	Object Assembly	27 (30%)	23 (37%)	22 (45%)
	Similarities	20 (22%)	17 (27%)	18 (37%)
Valley	Picture Completion	46 (52%)	15 (24%)	9 (18%)
	Information	21 (24%)	13 (21%)	7 (14%)
	Coding	12 (13%)	11 (17%)	16 (33%)

Data are presented as cases (n) or proportion (%). Due to the high and low scores of the scale being able to coexist across different subtests, the total proportions may exceed 100%. *HF-ASD* High-Functioning Autism Spectrum Disorder, *ADHD* Attention Deficit Hyperactivity Disorder, *ASD+ADHD* comorbidity.

### 3.5 Quantification of the peak-valley differences

Variance and quantitative analysis of subtest differences revealed significant differences between groups in intra- and inter-subtest scores (**Table 3**). The ASD + ADHD group had a mean peak-valley difference of 2.62 SDs in the intra - subtest and 2.94 SDs in the inter - subtest. The HF-ASD group had a mean difference of 2.69 SDs in the intra - subtest and 3.05 SDs in the inter - subtest. In contrast, the ADHD group had a mean difference of 1.79 SDs in the intra - subtest subtests and 2.07 SDs in the inter - subtest (**Table 3**). The ROC curve to validate 2.6SDs showed an AUC of 0.824.

**Table 3:** Comparison of the Peak-Valley Differences between Children with HF-ASD, ADHD,ASD+ADHD.

Difference	Name	Mean	Relationship with multiples of the standard deviation(*SD)	F	*p*
Intra- subtest Difference	ADHD	5.384	1.792	22.747	p<0.001**
	ASD+ADHD	7.873	2.623		
	HF-ASD	8.086	2.696		
Inter- subtest Difference	ADHD	6.213	2.071	24.092	p<0.001**
	ASD+ADHD	8.814	2.948		
	HF-ASD	9.162	3.057		

Intra- subtest Difference: The difference between the highest and lowest scores for each test item in the performance or verbal group; Inter- subtest Difference: The difference between the highest and lowest test scores for all projects; *HF-ASD* High-Functioning Autism Spectrum Disorder, *ADHD* Attention Deficit Hyperactivity Disorder, *ASD+ADHD* comorbidity; *p<0.05, **p<0.01.

## 4 Discussion

Previous studies have documented the cognitive strengths and deficits in the intellectual architecture of children diagnosed with ASD (26, 45, 46). However, no studies have systematically leveraged intellectual traits of ASD to differentiate it from ADHD. Thus, this study represents the first systematic investigation of intellectual constructs in differentiating ASD, ADHD and comorbid ASD+ADHD. Consistent with established epidemiological trends (47), our cohort exhibited a male predominance (76.6%–90.48%). Children with ASD+ADHD demonstrated significantly lower total IQ compared to pure ADHD or HF-ASD groups. We proposed that functional impairment is more severe in children with ASD+ADHD. These findings suggest that the comorbid group’s hybrid profile suggests additive neurodevelopmental disruptions, where ASD-related strengths coexist with ADHD-related inhibitory deficits, exacerbating functional imbalances (9). Research results revealed significant imbalance between VIQ and PIQ across groups, and further found that the VIQ of children with HF-ASD and comorbid ASD and ADHD was significantly lower than their PIQ by about 0.3 SDs corroborating prior findings (21, 26). VIQ reflects crystallized intelligence—a domain influenced by cultural and educational backgrounds—encompassing knowledge, vocabulary, language comprehension, and academic skills (48). However, PIQ represents fluid intelligence, relying on innate factors like perception, memory, processing speed, and reasoning abilities (49). These findings may be related to the fact that children with ASD develop later in life with impaired social participation and communication, which hinders the development of crystallized intelligence and leads to low VIQ (50, 51). ADHD group are more likely to be affected by deficits in innate abilities (fluid intelligence deficits) and less likely to be affected by later-developing abilities such as language (52, 53). Therefore, differences between VIQ and PIQ may help distinguish between ASD.

Children with HF-ASD or ASD+ADHD exhibited greater subtest score fluctuations compared to those with ADHD alone, with pronounced peak-valley discrepancies (54). These findings collectively suggest a markedly imbalanced intellectual structure in children with ASD, featuring distinct strengths and weaknesses. The results of the analysis of the proportion indicate that the peaks of the HF-ASD group and the Comorbidity group are mostly concentrated in the Block Design, whereas the ADHD group is more focused on the Object Assembly. This is similar to previous research reports (55). This difference may stem from the fact that while Block Design and Object Assembly both assess visuospatial abilities, the latter additionally demands adaptive visuomotor integration—a skill contrasting with the rigid, stereotypical mode of Block Design (20). Elevated visuospatial abilities in ASD (such as peak performance on Block Design) may reflect atypical neural specialization in right-hemispheric regions, particularly the dorsal visual stream responsible for spatial processing (15). Besides, the valleys of the HF-ASD group and the Comorbidity group are mostly focused on the Picture Completion, whereas the ADHD group showed significantly lower scores on Coding subtests (19). This phenomenon may be attributable to the notion that coding is indicative of working memory capacity. The findings that children diagnosed with ADHD exhibit impairments in working memory are consistent with the conclusions of previous studies (56). The Picture Completion has been demonstrated to reflect visuomotor dexterity and visual recognition of basic details of objects. Previous studies have shown that children diagnosed with ASD have deficits in this area (57). Consequently, the study proposed the cognitive strengths and weaknesses of HF-ASD,ADHD and comorbid ASD+ADHD can be differentiated through the C-WISC.

Building on prior research, this study extends the quantification of cognitive peak-valley discrepancies (intra- and inter-subtest score variations) in children with HF-ASD and ADHD, while exploring their neurocognitive underpinnings. Children with HF-ASD exhibited significantly greater intra- and inter-subtest variability, with mean peak-valley differences exceeding 3 SD—nearly 50% higher than the <2 SD observed in the ADHD group. These findings confirm that children with HF-ASD exhibited more pronounced peak-valley pattern—reflecting both exceptional strengths and marked deficits—relative to the weaker heterogeneity performance patterns of children with ADHD. Although not all individuals with HF-ASD exhibit significant peaks and troughs in their intellectual structure due to individual differences, this may be a subtype of ASD. However, from a clinical perspective, this phenomenon is quite common. While our findings align with earlier reports of uneven cognitive profiles in ASD (58, 59), they extend this work by bridging neuropsychological observations to clinical applications—a critical gap in prior research.

In the study, we wanted to look for a clinical link between this cognitive trait and further clues for identifying and diagnosing children with ASD and ADHD. In clinical practice, by calculating the difference between the highest and lowest scores on each subtest, if the difference exceeds 2.6 SD (SD = 3), it may indicate potential social issues, necessitating further evaluation using social-related scales and medical history for diagnosis. Of course, the possibility of underlying social difficulties cannot be ruled out. Certainly, due to the limited sample size of this study, this threshold requires further validation.

The cognitive strengths and weaknesses described above are specific manifestations of neurodiversity (24, 60). Both help doctors to take a medical history for early diagnosis, while enabling parents to better understand their children’s intellectual structure beyond academic scores (61). More importantly, we can also suggest different interventions for different cognitive characteristics. Leveraging the strengths of children with ASD can enhance their weaknesses, increase interaction through cultivating advantageous abilities (such as programming or chess), and foster social engagement and vocational readiness (62). Therefore, Paradigms for Identifying Cognitive Strengths and Weaknesses in children with ASD, ADHD and Comorbidity, especially those currently viewed as weaknesses but with potential for transformation, is significant. Thus, analysis of the structural imbalance features of intelligence, it can be demonstrated that children with ASD and ADHD have different cognitive paradigms, and combining this paradigm with clinical history can help physicians and families recognize children’s strengths and weaknesses in order to identify children with ADHD from those who have both autism and ADHD.

## 5 Limitations

Several limitations should be acknowledged. First, C-WISC may be affected by differences in cultural contexts and its application in other languages needs to be further validated, and it is not the newest scale, so may cause score bias and reduce sample representativeness compared to the WISC-IV/V. Besides, the sample was relatively small, the underrepresentation of females and ADHD children may limit the generalizability of cognitive findings, thereby leading to sample bias. And healthy populations and low-functioning cases (IQ<70) were not included in this study, which may limit applicability. The next step is to enrich the sample size in order to further refine the application of the cognitive paradigm in different populations. Finally, this study relied exclusively on clinical scales, which may introduce subjectivity and error, and could be combined with objective screening or diagnostic techniques (e.g., eye-tracking technology, near-infrared functional brain imaging, magnetic resonance, etc.) in the future.

## 6 Conclusion

This study used the C-WISC to analyze the intellectual structure of children between HF-ASD, ADHD, and ASD + ADHD. Notable imbalances were identified in the intellectual profiles of children with ASD and those with ASD + ADHD, characterized by a distinct peak-and-valley pattern with differences exceeding 2.6 SDs. Specifically, peaks were predominantly observed in the Block Design and Object Assembly subtests, whereas valleys were evident in the Picture Completion subtest. Although children with ADHD also exhibited peaks and valleys, the differences were smaller, typically under 2 SDs, with peaks mostly in Object Assembly and Similarities and valleys primarily in Coding.

These findings could help differentiate between HF-ASD, ADHD, and ASD + ADHD, and offer clinicians novel insights for diagnosing and intervening in ASD. By recognizing the cognitive strengths and weaknesses of children with ASD, clinicians and educators could devise personalized intervention strategies that cater to the unique needs of these children, providing a robust foundation for improving their outcomes.

## Data Availability

The data analyzed in this study is subject to the following licenses/restrictions: The datasets generated during and/or analyzed during the current study are available from the corresponding author on reasonable request. Requests to access these datasets should be directed to Tingyu Li, tyli@vip.sina.com.
