# Supplementary material for: The value of intellectual structural imbalance in the differentiation of autism spectrum disorder and attention deficit hyperactivity disorder
Source: Front Psychiatry. 2025 Aug 14;16:1610278. doi: 10.3389/fpsyt.2025.1610278 (PMC12391015; doi:10.3389/fpsyt.2025.1610278)
Supplement: Supplementary file 1 [file DataSheet1.docx]

Appendix Figure 1


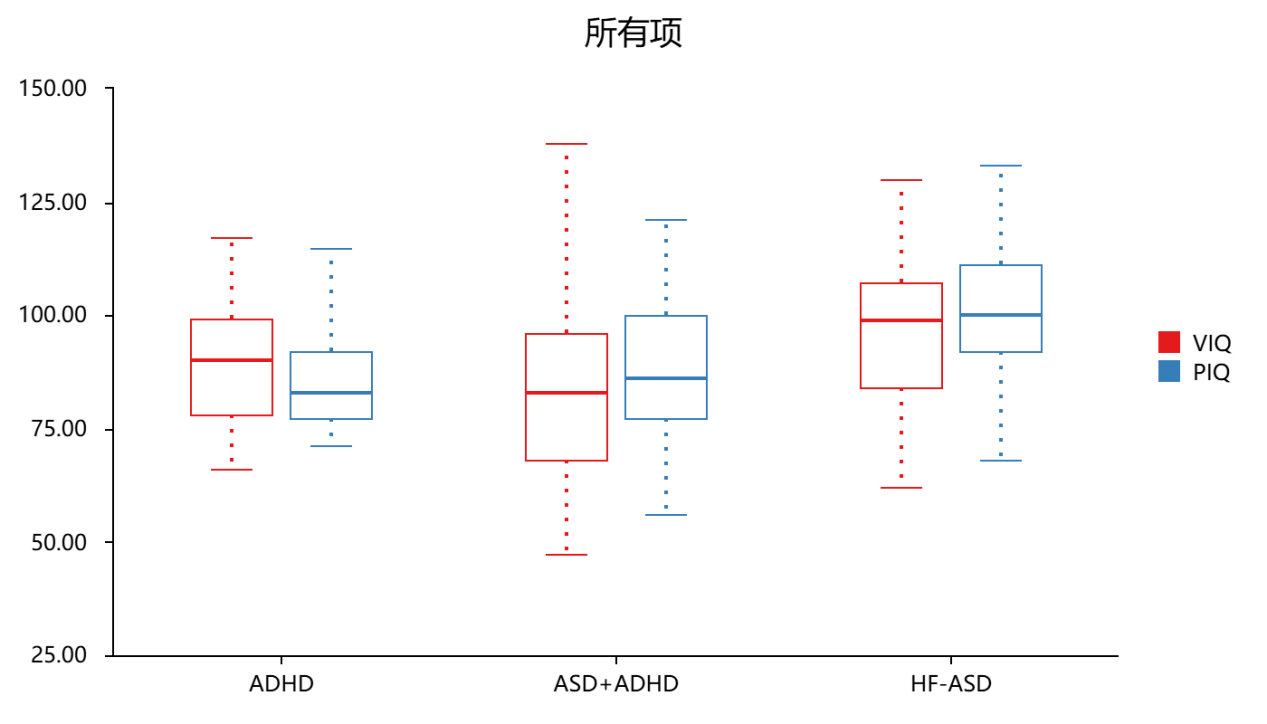


Fig. 1 Comparison of VIQ and PIQ between Children with HF-ASD、ADHD、ASD+ADHD

HF-ASD High-Functioning Autism Spectrum Disorder, ADHD Attention Deficit Hyperactivity Disorder, ASD+ADHD comorbidity.

Appendix Figure 2


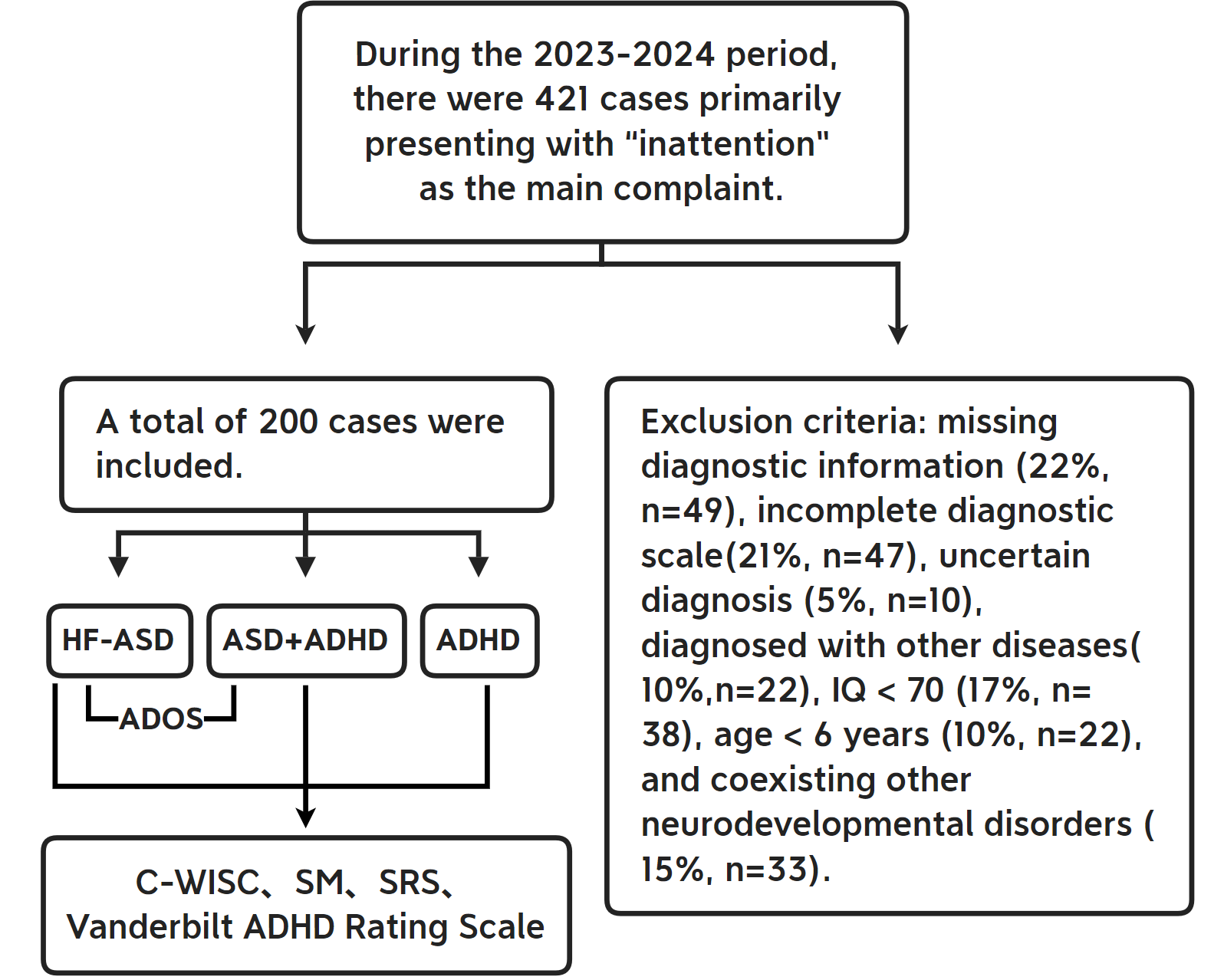


Fig. 2 Diagnostic grouping flow diagram of patients

*HF-ASD:H*igh-Functioning Autism Spectrum Disorder, *ADHD:A*ttention Deficit Hyperactivity Disorder, *ASD+ADHD* :comorbidity. *C-WISC* Chinese Wechsler Intelligence Scale for Children; *ADOS* Autism Diagnostic Observation Schedule; *S-M Scale* Infant-Junior High School Student Social Life Ability Scale; *SRS* Social Responsiveness Scale

Appendix Figure 3


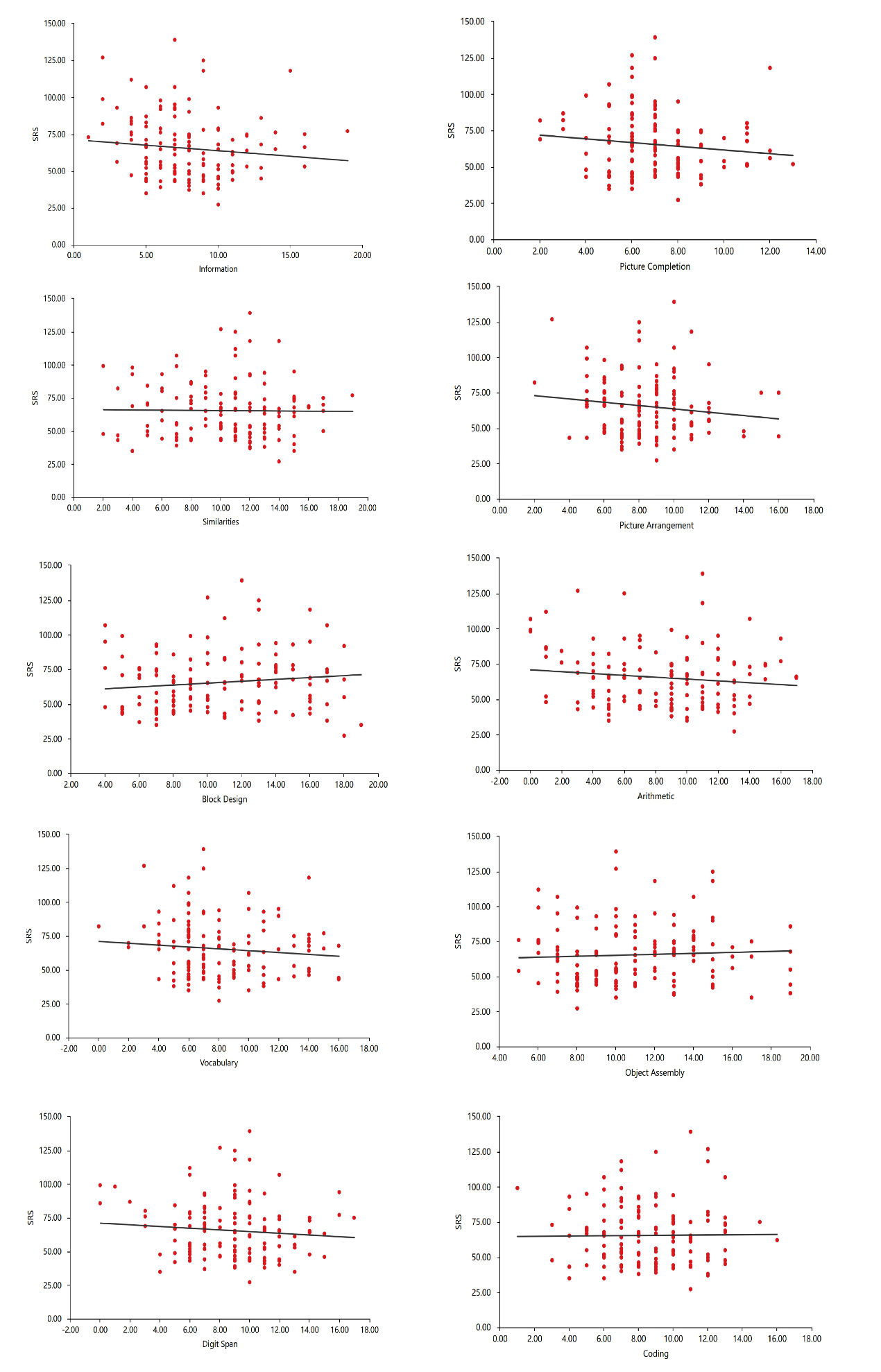


Fig. 3 Relationship between scores on the SRS scale scores and subtest scores

HF-ASD High-Functioning Autism Spectrum Disorder, ADHD Attention Deficit Hyperactivity Disorder, ASD+ADHD comorbidity.

Appendix Table 1

Differential analysis of Autism Diagnostic Observation Schedule (ADOS)

between HF-ASD and ASD+ADHD

| Name | Diagnostic Assessment (Mean ± Standard Deviation) | | F | p |
| --- | --- | --- | --- | --- |
|  | ASD+ADHD | HF-ASD |  |  |
| Communication Total Score | 3.002±1.751 | 2.470±1.914 | 1.249 | 0.268 |
| Social Interaction Total Score | 7.342±2.306 | 5.933±2.614 | 4.834 | 0.032* |
| Communication + Social Interaction | 10.341±3.730 | 8.402±4.211 | 3.525 | 0.066 |
| Play | 0.903±0.724 | 0.739±0.838 | 0.648 | 0.424 |
| Stereotyped Behaviors and Restricted Interests | 0.623±1.152 | 0.275±0.648 | 2.163 | 0.147 |

*HF-ASD H*igh-Functioning Autism Spectrum Disorder, *ADHD A*ttention Deficit Hyperactivity Disorder, *ASD+ADHD* comorbidity, **p<0.05* , ***p<0.01*

Appendix Table 3

Kruskal-Wallis testing for various subtests

| Name | Diagnostic Median(P_25_，P_75_) | | | *H*  | *p* |  |
| --- | --- | --- | --- | --- | --- | --- |
|  | ADHD | ASD+ADHD | HF-ASD |  |  |  |
| Information | 7.000(5.0,9.0) | 6.000(4.0,8.0) | 8.000(6.0,10.0) | 15.697 | P<0.001 |  |
| Similarities | 10.000(6.0,11.0) | 8.000(6.0,11.0) | 11.000(9.0,13.0) | 17.053 | P<0.001 |  |
| Arithmetic | 8.000(5.0,11.0) | 6.000(2.0,9.0) | 10.000(6.8,12.0) | 28.837 | P<0.001 |  |
| Vocabulary | 7.000(6.0,9.0) | 6.000(5.0,9.0) | 8.000(7.0,11.3) | 17.325 | P<0.001 |  |
| Digit Span | 9.000(6.0,10.0) | 7.000(5.0,10.0) | 10.000(8.0,12.0) | 25.571 | P<0.001 |  |
| Picture Completion | 7.000(6.0,8.0) | 6.000(5.0,7.0) | 7.000(6.0,8.0) | 17.092 | P<0.001 |  |
| Picture Arrangement | 8.000(7.0,9.0) | 7.000(6.0,9.0) | 9.000(7.0,10.0) | 20.246 | P<0.001 |  |
| Block Design | 7.000(6.0,9.0) | 10.000(7.0,13.0) | 13.000(9.0,15.3) | 52.526 | P<0.001 |  |
| Object Assembly | 9.000(8.0,10.0) | 10.000(8.0,14.0) | 12.000(9.0,14.3) | 20.520 | P<0.001 |  |
| Coding | 7.000(6.0,9.0) | 8.000(6.0,10.0) | 9.000(8.0,12.0) | 25.790 | P<0.001 |  |

*HF-ASD H*igh-Functioning Autism Spectrum Disorder, *ADHD A*ttention Deficit Hyperactivity Disorder, *ASD+ADHD* comorbidity, **p<0.05* , ***p<0.01*

Appendix Table 4

Dunn's t testing for various subtests

|  | Diagnostic | Median | Median | Difference | *p* |
| --- | --- | --- | --- | --- | --- |
| Information | ASD+ADHD | 7.000 | 6.000 | 1.000 | 0.118 |
|  | HF-ASD | 7.000 | 8.000 | -1.000 | 0.055 |
|  | HF-ASD | 6.000 | 8.000 | -2.000 | P<0.001 |
| Similarities | ASD+ADHD | 10.000 | 8.000 | 2.000 | 0.649 |
|  | HF-ASD | 10.000 | 11.000 | -1.000 | P<0.001 |
|  | HF-ASD | 8.000 | 11.000 | -3.000 | P<0.001 |
| Arithmetic | ASD+ADHD | 8.000 | 6.000 | 2.000 | 0.015* |
|  | HF-ASD | 8.000 | 10.000 | -2.000 | 0.022* |
|  | HF-ASD | 6.000 | 10.000 | -4.000 | P<0.001 |
| Vocabulary | ASD+ADHD | 7.000 | 6.000 | 1.000 | 0.172 |
|  | HF-ASD | 7.000 | 8.000 | -1.000 | 0.023* |
|  | HF-ASD | 6.000 | 8.000 | -2.000 | P<0.001 |
| Digit Span | ASD+ADHD | 9.000 | 7.000 | 2.000 | 0.238 |
|  | HF-ASD | 9.000 | 10.000 | -1.000 | P<0.001 |
|  | HF-ASD | 7.000 | 10.000 | -3.000 | P<0.001 |
| Picture Completion | ASD+ADHD | 7.000 | 6.000 | 1.000 | 0.025* |
|  | HF-ASD | 7.000 | 7.000 | 0.000 | 0.171 |
|  | HF-ASD | 6.000 | 7.000 | -1.000 | P<0.001 |
| Picture Arrangement | ASD+ADHD | 8.000 | 7.000 | 1.000 | 0.130 |
|  | HF-ASD | 8.000 | 9.000 | -1.000 | 0.016* |
|  | HF-ASD | 7.000 | 9.000 | -2.000 | P<0.001 |
| Block Design | ASD+ADHD | 7.000 | 10.000 | -3.000 | P<0.001 |
|  | HF-ASD | 7.000 | 13.000 | -6.000 | P<0.001 |
|  | HF-ASD | 10.000 | 13.000 | -3.000 | P<0.001 |
| Object Assembly | ASD+ADHD | 9.000 | 10.000 | -1.000 | 0.040* |
|  | HF-ASD | 9.000 | 12.000 | -3.000 | P<0.001 |
|  | HF-ASD | 10.000 | 12.000 | -2.000 | P<0.001 |
| Coding | ASD+ADHD | 7.000 | 8.000 | -1.000 | 0.555 |
|  | HF-ASD | 7.000 | 9.000 | -2.000 | P<0.001 |
|  | HF-ASD | 8.000 | 9.000 | -1.000 | P<0.001 |

*HF-ASD H*igh-Functioning Autism Spectrum Disorder, *ADHD A*ttention Deficit Hyperactivity Disorder, *ASD+ADHD* comorbidity, **p<0.05* , ***p<0.01*

Appendix Table 5

paired t-testing between VIQ and PIQ

| Name | Paired Groups (Mean ± SD) | | Difference (Pair 1 - Pair 2) | *t* | *p* |
| --- | --- | --- | --- | --- | --- |
|  | Pair 1 | Pair 2 |  |  |  |
| VIQ(ADHD)   vs.   PIQ(ADHD) | 90.43±13.95 | 85.51±9.83 | 4.91 | 2.413 | 0.020* |
| VIQ((ASD+ADHD）   vs.   PIQ(ASD+ADHD） | 83.15±19.58 | 88.54±14.70 | -5.39 | -2.632 | 0.011* |
| VIQ（HF-ASD）   vs.   PIQ（HF-ASD） | 96.62±16.59 | 100.82±12.76 | -4.21 | -2.553 | 0.012* |

*HF-ASD H*igh-Functioning Autism Spectrum Disorder, *ADHD A*ttention Deficit Hyperactivity Disorder, *ASD+ADHD* comorbidity, V*IQ* verbal intellectual quotient, P*IQ* performance intellectual quotient, **p<0.05* , ***p<0.01*

Appendix Table 6

Analysis of ROC results

| Name | AUC | Standard error | p | 95% CI |
| --- | --- | --- | --- | --- |
| Inter-subtest Difference | 0.824 | 0.033 | 0.000** | 0.759 ~ 0.889 |

**p<0.05* , ***p<0.01*

Appendix Table 7

Partial correlation analysis of SRS scores and Block Design

|  | mean | standard deviation | SRS | Block Design |
| --- | --- | --- | --- | --- |
| SRS | 66.034 | 21.223 | 1 |  |
| Block Design | 10.438 | 3.944 | 0.237** | 1 |
| Total IQ | / | / | -0.107 | 0.676** |
| Verbal IQ |  |  | -0.125 | 0.458** |
| Age |  |  | 0.052 | 0.103 |

*SRS* Social Responsiveness Scale, **p<0.05* , ***p<0.01*
